# A Case of Heart Disease

**Published:** 1849-08

**Authors:** David W. Yandell

**Affiliations:** Louisville, Kentucky


Art. V.—*A Case of Heart Disease.* By David W. Yandell, M. D., of Louisville, Ky.

Through the kindness of a professional friend of this city, I have been furnished with the following brief outline of a very remarkable case of disease of the heart. The subject was a negro man, thirty-five years of age, of good constitution, but intemperate habits.

Towards the close of December last, while recovering from an attack of cholera, his superior and inferior extremities and face, particularly the latter, became edematous, and he suffered almost total loss of vision. Under the influence of mercury, the edema slowly but gradually disappeared, and his sight was partially restored.

In April, when my friend saw him for the first time, his extremities and face were infiltrated ; his eyes considerably protruded ; conjunctivas distended with serum ; sight entirely gone, and he lay in a comatose condition, from which he was with great difficulty aroused. On applying the ear over the precordial region, the impulse of the heart was found to be distinct and strong, and accompanied by a bellows sound, audible over the whole of the organ, apparently most distinct at its base. Pulse strong, full, and not accelerated. Ordered a large venesection, followed by calomel and jalap; under which treatment the cerebral symptoms speedily amended: Mercury, guarded by opium, was administered, with a view of touching the gums, which had been effected but a short time when the bellows sound sensibly diminished, the edema disappeared, and his sight became better. The patient complaining of great weakness, was put upon tonics and allowed generous diet, which, for a time, gave promise of effecting a cure ; but, finally, without a return of the edema, with almost complete restoration of vision, the patient died apparently from sheer exhaustion. Owing to certain circumstances, the heart alone was examined. It was found somewhat hypertrophied, being three or four ounces heavier than the average; its left side healthy; the walls of the right side and the tricuspid valves slightly thickened, and the cavities dilated; whole internal surface of the pericardium was covered with a thin stratum of lymph, most evident at its lower part, and a few flakes of lymph were found floating in a small quantity of serum.

Every reader of the above will concur with me in the regret that only so brief and imperfect a history of this case has been preserved. It is, so far as I know, unique in the annals of science.

Notwithstanding the many and able treatises which have been written on the subject, the diseases of the heart are probably less understood generally, in this country, than any other class of affections. They are diseases that more than any other require to be studied at the bedside, and even here it is desirable, if not essential, to have the guidance and instruction of some one familiar with their manifestations. I deem it unfortunate that Dr. Smith has seen fit, in his translation of Barth & Roger's Manual on Auscultation and Percussion, to omit the table of signs of diseases of the heart contained in the original. He would have laid the profession under still greater obligations to him had he prepared a table of these signs as extensive and accurate as he has one of those of the diseases of the respiratory organs.

With the view of partially supplying this deficiency I herewith offer the table alluded to, translated from the original, and will afterwards add another that I hope will not be entirely without merit.

3

### A TABLE OF THE PATHOLOGICAL SIGNIFICATION OF THE BRUITS DE SOUFFLE.

| | | | |
|---|---|---|---|
| *Souffle* before the first sound. | - - - - | - - - - | *Auriculo-ventricular narrowing.* |
| *Souffle* accompanying the first sound. | Arterial narrowing, or | Maximum at the base of the heart, with propagation into the large arteries. | *Arterial narrowing,* |
| | Auriculo-ventricular insufficiency. | Maximum towards the point of the heart, without propagation into the large arteries. | *Auriculo-ventricular insufficiency.* |
| *Souffle* accompanying the second sound. | Arterial insufficiency, or | Maximum at the base of the heart, with propagation into the large arteries. | *Arterial insufficiency.* |
| | Auriculo-ventricular narrowing. | Maximum at the point of the heart, without propagation into the large arteries. | *Auriculo-ventricular narrowing.* |
| *Souffle* after the second sound. | - - - - | - - - - | *Aneurism of the Aorta.* |

*Barth & Roger, p.* 434.

It will be remarked that the following table, as that of MM. B. & R., embraces alone the diseases of the valves and orifices of the heart.

A SYNOPTICAL TABLE OF ABNORMAL SOUNDS HEARD IN DISEASES OF THE VALVES AND ORIFICES OF THE HEART.

| | | | |
|---|---|---|---|
| *Souffle* accompanying the first sound. | Corresponding to the more or less violent impulse of the point of the heart against the thoracic wall;—more or less short, rude, rasping, sometimes whining; absence of the valvular *clacking;* having its maximum at the point; vibratory thrill more or less marked, sometimes synchronous with the impulse of the point of the heart against the thoracic wall and with the arterial pulsation. | Maximum at the point and outside of the left nipple; valvular clack of the first sound preserved along the sternum;—pulse soft, easily compressed; frequent pulmonary hemorrhages. | Insufficiency of the auriculo-ventricular orifice of the left side (consequent upon adhesions or more or less deformation. |
| | | Maximum at the base of the sternum and point of the heart extending also along the whole length of the sternum, with absence of the valvular *clacking* in these regions, while it is heard on the left; pulse soft, compressible; speedy general infiltration, ascites; regurgitation of blood in the auricles; distension of jugulars, &c.; disposition to cerebral apoplexies. | Insufficiency, by adhesion or deformation more or less complete of the auriculo-ventricular orifice of the right side, (less frequent than the preceding.) |
| | Corresponding to the impulse of the point of the heart, which is very strong, having its maximum at the base; preservation of first valvular *clacking ;* generally prolonged, covering in part or entirely the short silence, and encroaching sometimes upon the second sound; frequent vibratory thrill; pulse small and generally hard, irregularity and intermissions in the sounds of the heart and in the pulse. | Maximum at the base of the heart, propagated into the large arteries; frequent pulmonary hemorrhages; pulse hard, irregular and intermittent. | Narrowing of the arterial orifice. |
| | | Maximum at the base; regurgitation in the jugular and communicating veins; general infiltration; pulse small and soft; disposition to cerebral apoplexies. | Narrowing of the orifice of the pulmonary artery (extremely rare.) |

| | | | |
|---|---|---|---|
| *Souffle* accompanying the second sound. | Corresponding to the depression of the point of the heart, and consequently to the ventricular diastole; more or less short; with absence of the valvular *clacking*, having itsmaximum at the base; disposition to general edema; sometimes a vibratory thrill synchronous with the diastole. | Absence of valvular *clacking* on the left; *gentle souffle*, disposition to infiltration and to pulmonary apoplexies; pulse bounding, elevated, soft, easily compressed. | Insufficiency by adhesions, or more or less complete deformation of the sigmoid valves of the aorta. |
| | | Absence of the valvular *clacking* of the second sound at the superior part of the sternum; regurgitation of the jugular veins, &c. | Insufficiency of the sigmoid valves of the pulmonary artery (this is very rare—there are only a few examples on record.) |
| | Corresponding to the depression of the point of the heart, and consequently to the ventricular diastole, prolonged *souffle*, occupying almost the whole of the long silence and sometimes encroaching upon the first sound of the next beat; rude, resembling the saw sound; valvular *clacking* preserved; maximum at the point; pulse small and soft; distension of the auricles; forming with the second normal sounds a triple sound in the following order: tic . . . tac . . . f-f-f . . . | Maximum at the point of the heart; intense; outside the left nipple; pulse small, soft; disposition to pulmonary apoplexies; inaudible or heard at least very feebly at the base of the heart and over the sternum. | Narrowing of the auriculo-ventricular orifice of the left side. |
| | | Maximum over the sternum and especially at the base; distension of the jugulars and communicating veins; regurgitation in these vessels; disposition to general infiltration; face injected; disposition to cerebral apoplexies; pulse developed and soft. | Narrowing of the auriculo-ventricular orifice of the right side (very rare. |

Before dismissing the subject of diseases of the heart, I have thought that the subjoined case which came under my observation a short time since at the Louisville Marine Hospital, might be read with interest as a striking example of one of the diseases under consideration.

Mr. S. S., æt. 40 years, a native of Massachusetts, a hatter, of bilious temperament, sober habits and good constitution, was admitted into the hospital on the 7th of July. Fifteen weeks before he was treated in the city for an attack of acute rheumatism which supervened upon exposure to cold. It affected principally the smaller joints and confined him to his bed for twenty-one days. He now recommenced his work though still suffering with sharp pains in the shoulders, superior part of the chest, elbow and wrist joints and fingers, which two last were swollen and very stiff. This condition of things becoming daily more and more aggravated he was obliged to give up work and enter the hospital.

*Condition at the time of Admission.*—Surface of the body pale, of the natural warmth and moisture. Countenance expressive of pain. Pain in the parts above mentioned, particularly in the superior part of the chest, precordial region and neck. Finger joints still somewhat swollen and stiff. Appetite good, tongue clean, bowels regular. Rests well at night. Pulse natural. Limits of the heart upon percussion normal. On applying the ear over this organ its sounds appear confused: impulse and rythm natural. A distinct bellows murmur after the first sound was perceptible over its whole extent; at the sigmoid valves the sound assumed the character of the gentle friction sound which was propagated along the aorta to its arch. The peculiarity in this friction sound was its strong resemblance to crepitation. The valvular *clacking* of both sounds of the heart was normal.

*Diagnosis.*—Effusion of lymph, which at some points has been organized and contracted adhesions over the whole internal surface of the pericardium; deformation of the aortic valves, both resulting from previous endo and exocardial inflammation.

*Treatment.*—The patient was ptyalized; constant counter irritation set up over the heart, and Dover's powder in combination with the nitrate of potash administered with the view of their anodyne and diaphoretic effects. This treatment was pursued for two weeks; at the end of the first week the bellows murmur was less loud and distinct than before; friction sound the same; pain much less.

On the 21st, eight days after this time, the patient left the hospital to resume his work. The pain had almost entirely disappeared. The friction sound was undiminished; the bellows murmur was evidently much less intense though still clearly perceptible; the impulse and sounds of the heart were materially the same as at the time of his admission.

It need hardly be said that Mr. S. left the hospital much against my will, but urging that he had a wife and children who depended on his labors, I could not refuse his request although I felt that he was still unfit to renew his somewhat laborious profession. I prescribed for him at the time of his discharge, c. syrup sarsaparill. oj. and potass. hydriodat. ℥i.: M. Dose, two tablespoonsful three times aday.

I cannot do better than conclude the present article with the remarks of MM. Barth & Roger on the pathological signification of the bellows and friction sound.

The sounds which accompany organic lesion of the heart are sometimes *soft*, but more frequently *rough*, and resemble *rasping*, *filing sounds*, &c. On the other hand, *blowing sounds without* material lesion are in the majority of caseses *very soft*. The *former* accompany either the *first or second sound* of the heart; the latter are always heard during the *first*, and *never during the second*. The former are *constant*, and last for *months* or *years;* the latter are *intermittent* and *transient*. The *former* undergo progressive transformation from the *gentle souffle* to the *musical sounds*, in proportion to the length of time they exist, and as the lesions of the orifices become more *extensive* and *deeper seated*, the latter uniformly preserve their character of softness, whatever may be their modification of intensity.

Finally, *the rough sounds* are accompanied by local and general symptoms characteristic of disease of the heart, (dulness on percussion, a purring sound, irregularities of the pulse, considerable edema of the inferior extremities,) whilst none of these phenomena show themselves either in anemia or chlorosis, at least, in a decided or persistent manner.

The existence of a material lesion being admitted, the next point is to discover its nature. The souffles indicative of pericarditis, hypertrophy, the formation of a clot in the cavities of the heart, &c., are accompanied by peculiar symptoms, such as—prominence and dulness of the precordial region, diminution of impulse with feebleness and distance of sounds (pericarditis,)—dulness, increase of intensity in the sounds and impulse (hypertrophy,)—the sudden appearance of an abnormal sound with smallness of the arterial pulse (formation of clots.) These lesions being well marked by their diagnostic signs, there remain only the diseases of the orifices and valves, and in the consideration of their principal effects these may be arranged under two heads, *narrowings* and *insufficiencies*.

How shall we discover whether there be *narrowing* or *insufficiency?* To reply to this question we must first examine the *time* to which the abnormal sound belongs, determine whether it precedes or accompanies the systole, or whether it coincides with the diastole of the heart, and we can deduce from it the morbid signification of the souffle, by recalling what takes place at each of these movements. Now reason and experience demonstrate that *a souffle accompanying the first sound may indicate either a narrowing of the arterial orifices*, (causing increased friction of the blood in its onward progress,) or *an insufficiency of the auriculo-ventricular opening*, (which also gives rise to friction during the reflux of the columns of the blood.)—On the other hand, *a souffle accompanying the*

*second sound, indicates arterial insufficiency*, or, more rarely, *an auriculo-ventricular narrowing. The sign of this last alteration, is frequently a pre-systolic souffle.*

*The precise determination of the situation of the diseased orifice will now decide what is the nature of the lesion.*

If we decide, for example, that there exists a lesion of the arterial orifice, in a case where the souffle replaces the first sound of the heart, we shall have diagnosticated an arterial narrowing.

But the seat of disease may be discovered by an accurate knowledge of the place where the maximum of the *souffle* is produced, and by considering whether it is extended into the large vessels, or whether it is heard only around the region of the heart. In fact, the souffle dependent upon a lesion of the sigmoid valves, has its maximum intensity above the nipple at the base of the heart, and may extend into the large arteries, whilst that dependent upon alteration of the auriculo-ventricular valves, has its maximum below the nipple, nearer the point of the heart, and does not extend into the great arterial trunks.

If, then, a souffie accompanying the *first sound* has its *maximum intensity* at the *base of the heart*, and *extends into the large arteries*, it will indicate *arterial narrowing*. A souffle accompanying the first sound which has, on the contrary, its *maximum* at the *point* of the organ, *without* extending into the great arterial trunks, will indicate *auriculo-ventricular insufficiency*. On the other hand, a souffle accompanying the *second sound*, having its *maximum intensity above the nipple*, and *extending* into the *great vessels*, will announce *insufficiency of the sigmoid valves;* whilst the same sound, if it should have its *maximum below the nipple without* extension into the great arterial trunks, will diagnosticate *auriculo-ventricular* narrowing.

